# “We help people change harmful norms”: Working with key opinion leaders to influence MNCH+N behaviors in Nigeria

**DOI:** 10.1371/journal.pone.0308527

**Published:** 2024-08-15

**Authors:** Adetayo Adetunji, Eno-Obong E. Etim, Mayokun Adediran, Alessandra N. Bazzano

**Affiliations:** 1 Population Council, Abuja, Nigeria; 2 Department of Social, Behavioral, and Population Sciences, Center of Excellence in Maternal and Child Health, Tulane University School of Public Health and Tropical Medicine, New Orleans, Louisiana, United States of America; Universite de Parakou, BENIN

## Abstract

**Background:**

**Nigeria’s** Maternal, newborn, and child health and nutrition (MNCH+N) outcomes rank among the world’s poorest. Engaging traditional and religious leaders shows promise in promoting related behaviors. The Breakthrough ACTION/Nigeria project worked with leaders in northern Nigeria to implement the Advocacy Core Group (ACG) model, a social and behavior change (SBC) approach aimed at influencing community norms and promoting uptake of MNCH+N behaviors. Qualitative assessment of the model contributes to evidence on SBC approaches for enhancing integrated health behaviors.

**Methodology:**

This qualitative study was conducted in Nigeria’s Bauchi and Sokoto states in May 2021. It involved 51 in-depth interviews and 24 focus group discussions. The study was grounded in the social norms exploration (SNE) technique to examine normative factors influencing behavior change within the ACG model context. Data analysis used a reflexive thematic analysis approach. Ethical approvals were received from all involved institutions and informed consent was obtained from participants.

**Results:**

The ACG model was vital in the uptake of MNCH+N behaviors. The influence of ACG members varied geographically with greater impact observed in Sokoto State. Normative barriers to improving MNCH+N outcomes included perceived religious conflicts with family planning, preference for traditional care in pregnancy, misinformation on exclusive breastfeeding (EBF), and gender-based violence resulting from women’s decision-making. The study demonstrated positive progress in norm shifting, but EBF and GBV norms showed slower changes. Broader challenges within the health system, such as inadequate services, negative attitudes of healthcare providers, and workforce shortages, hindered access to care.

**Conclusion:**

The ACG model increased awareness of health issues and contributed to potential normative shifts. However, slower changes were observed for EBF and GBV norms and broad health system challenges were reported. The model appears to be a promising strategy to further drive SBC for better health outcomes, especially where it is combined with supply-side interventions.

## Background

Over the past three decades, maternal and newborn mortality has decreased globally, nevertheless, low- and middle-income countries continue to disproportionately experience the highest rates [1]. Maternal, newborn, and child health and nutrition (MNCH+N) outcomes in Nigeria continue to be among the worst in the world despite series of interventions [2–4]. This is further evidenced by relevant indicators as captured by the 2018 Nigeria Demographic and Health Survey as well as comparison of these indicators to that of other countries on global tracking dashboards such as the countdown to 2030 dashboard supported by global and regional institutions including UN agencies [5, 6]. Expanding access to MNCH+N related services in Nigeria remains a public health concern. This is especially the case in northern Nigeria where communities are patriarchal and men are often sole decision makers [7–10]. Studies have shown that male partners may decide if, when and where a woman may access health care, and whether or not to provide financial resources to travel to receive these services [11, 12]. However, men who hold decision-making power are not necessarily knowledgeable about pregnancy and childbirth-related complications [13, 14]. This notion has often necessitated ideations about the involvement of men in MNCH+N processes to ensure positive outcomes. This is evidenced by a study that explored the experience of men who participated in initiatives to promote the demand for MNCH services in Northern Nigeria where men who participated in this process internalised important messages learnt and has led to a cascade of knowledge in the community leading to increased utilisation of services once more validating the important role of men in MNCH [15]. This was corroborated by a study on male involvement in maternal health in Adamawa state that found that while male involvement is key to better maternal outcomes, religious and cultural factors as well as ignorance limit men from participating in maternal health care [16]. Other studies that found poor knowledge of associated maternal health danger signs suggested addressing this gap through targeted male involvement in maternal health considering potential benefits [17].

Barriers to MNCH+N behaviors include specific norms favoring non-facility-based childbirth, such as a preference for natural childbirth processes, and modesty in northern Nigeria where only a woman’s husband is allowed to see her unclothed body [18]. Misinformation about the benefits of colostrum also continues to drive suboptimal rates of EBF [19–21]. Family planning uptake in northern Nigeria has also been shown to be negatively influenced by the association of large families with economic benefits and social advantages of multiple children signaling wealth and high status [22–25], Additionally, religious beliefs that emphasize God’s command to procreate and discourage the need for birth control contribute to the reluctance in limiting childbearing [26–28]. These social norms, combined with other sociocultural factors such as skepticism of western public health interventions have resulted in the emergence of narratives that discourage the uptake of reproductive health, immunization, and nutrition programmes [29, 30].

Several studies have highlighted the importance of addressing socio-cultural barriers and norms to improve the uptake of MNCH+N services [31, 32]. Addressing these barriers involves leveraging influential personalities within communities such as religious and traditional leaders. In northern Nigeria, traditional and religious leaders are highly influential in all aspects of people’s lives [33]. This influence often extends to health-related issues, particularly related to reproductive function and fertility, issues that are often assumed to be “up to God” [23]. Studies in Nigeria and other West African countries have identified a potential role for religious leaders in improving health and shifting socio-cultural norms that influence MNCH+N behaviors [34–36], although thus far, programs using this approach have been principally focused on family planning.

The USAID-funded Breakthrough ACTION/Nigeria project has been working with religious and community leaders in northern Nigeria since 2017 to implement the Advocacy Core Group (ACG) model. The ACG is comprised of religious and traditional leaders who wield influence in communities. Breakthrough ACTION/Nigeria is focused on improving priority MNCH+N behaviors through 1) advocacy outreach to opinion leaders and community influencers at State and LGA levels; 2) direct community engagement through dialogues and meetings that include referrals for services; and 3) SBC messaging through mass and mid-media. Utilizing the ACG approach, Breakthrough ACTION/Nigeria offers significant capacity building and technical support concerning resource mobilization, program design, execution, monitoring, evaluation, and more to ACG members. This empowers them to effectively convey health-promoting messages, ultimately aiming to reshape local norms, drive service utilization, and foster behaviors related to MNCH+N. The support provided through the ACG approach is expected to further aid ACG members in implementing their diverse responsibility, encompassing dispelling misunderstandings and obstacles related to MNCH+N initiatives; fostering demand and promoting the uptake MNCH+N services, including family planning and malaria, while also advocating for resources from governments, NGOs, and institutions; collaborating with local leaders, challenging detrimental norms, and raising related awareness at state and local levels through social and behavior change communication channels to facilitate effective uptake of MNCH+N related behaviors; and strengthening access to high-quality MNCH+N services by establishing robust connections between communities and healthcare facilities, with the goal of enhancing MNCH+N outcomes.

To qualitatively assess the ACG model, this paper draws on a study conducted by Breakthrough RESEARCH/Nigeria and explores how the model, as an integrated health platform, operates, and its potential effectiveness in influencing community-level norms and individual behaviors for MNCH+N, family planning, and malaria prevention. Breakthrough RESEARCH was an SBC research and evaluation initiative that functioned as a sister project to Breakthrough ACTION, providing critical SBC evidence to support health programming. It is intended that the findings from this study will contribute towards the broader SBC implementation science literature surrounding the roles, effectiveness, successes, and challenges of leveraging religious and traditional leaders and social structures to improve social normative environments for health. This study aligns with country priority efforts to improve MNCH+N outcomes.

## Methods

### Study design

The study was qualitative in design and included 51 in-depth interviews (IDIs) and 24 focus group discussions (FGDs) that were designed using the social norms exploration (SNE) approach [37]. The SNE technique helped to better understand facilitators and barriers to behavior change in the context of the ACG model. The SNE technique is a participatory approach used to uncover social norms that impact target behaviors of interest with its findings shaping the intervention design and monitoring for results [37, 38]. Furthermore, it is primarily a qualitative, team-based rapid framework that lays emphasis on key concepts such as social norms particularly injunctive and descriptive norms, reference groups, and behaviors of interest. It also asks key questions such as *“Which groups are most influential to the Main Population Group regarding the behavior of interest*?*”*, *“What are the social norms that influence this behavior*?*”*, *“Why do people comply with social norms*? *or Why not*?*”*, and *“What are the social norms that influence this behavior the most*?*”*

We adapted the SNE steps as provided by the Institute for Reproductive Health at Georgetown University which include planning, identification of reference groups, exploration of social norms, analysis, and application of findings [37]. These steps are further described below.

The SNE planning phase allows for reflection on social norms that influence the behaviors of interest. It also helps identify and segment the primary population. Furthermore, this phase allows us to establish exploration goals, create activities, decide the number of communities and individuals to engage, and plan for fieldwork. During this phase of the study, we identified 17 important health behaviors based on the Breakthrough ACTION Nigeria intervention. We also identified the study’s participants as program beneficiaries and ACG members.

Following the planning phase, the next stage is to determine reference groups. This entails interacting with potential participants to learn who they seek advice from and who impacts their behavior. Specialized exercises, such as the ’My Social Networks’ speed interview sessions, are utilized to assist participants in identifying significant persons associated with their preferred habits. In this study, people identified religious and traditional leaders, including members of the ACG, as major influencers.

Subsequently, the third phase which involves the exploration of social norms sees the research team further engage participants this time in addition to program beneficiaries and ACG members who were identified as the reference group in the previous phase. In this phase, we utilized vignettes to explore normative factors and their influence on behaviors of interest. We also incorporated the ‘Five Whys’ exercise, a problem-solving technique used to explore the root cause of an issue by repeatedly asking the question "Why?"

The fourth phase included data analysis utilizing a reflexive thematic analysis method. In addition, we conducted a participatory rapid analysis, specifically for the Five Whys exercise. This process identified major factors influencing behaviors of interests and compared program beneficiaries’ viewpoints to those of ACG members.

Finally, the findings that emanate from the study is provided to Breakthrough ACTION Nigeria team to further adjust components of the SBC-ACG interventions for suitable results.

While our application of the SNE methodology primarily centered on participants’ perspectives on social norms and behaviors, conducting IDIs specifically with ACGs also provided insights into the operational aspects of the ACG model including their roles, activities, and challenges.

### Study area

The study was implemented in the Nigerian states of Bauchi and Sokoto. These states were selected as they are implementation states for the Breakthrough ACTION/Nigeria project. Bauchi State has a population of 7,788,504 [39] people and study LGAs in Bauchi state included Bauchi, Ganjuwa, and Misau. Sokoto state has an estimated population of 6,039,289 people [39] and study was conducted in Wammako, Dange Shuni, and Kware LGAs of Sokoto state. MNCH+N-related indicators such as modern contraceptive use and health facility delivery are poor across the two selected states [5].

### Study population

The study utilized purposive sampling to select two categories of participants namely, ACG members and program beneficiaries. ACG members included religious; women; and traditional leaders, who were living in the communities, and were active members of the group. The community members were men, and women of reproductive age (15–49 years) who were caregivers of under-2 children, and beneficiaries of the ACG intervention. The inclusion criteria for both groups of participants are listed below.

#### Inclusion criteria

**ACG members**
  1. Must be an active ACG member.
  2. Live in the same community in which the ACG work takes place.
  3. Must provide informed consent.
**Program beneficiaries**
  1. Women aged 15 to 49 years who are currently pregnant or parent/caregiver of a child under 2 years.
  2. Men aged 15 to 49 years who are currently parents/caregivers of a child under 2 years.
  3. Has been a beneficiary of the ACG intervention.
  4. Must provide informed consent.

### Data collection process

#### Study tools

The FGD guides focused on identifying key influencers/decision-makers for healthcare decision-making within households and communities, social norms that shape gender roles, and behaviors of interest within communities; utilizing vignettes, or hypothetical stories related to the behaviors of interest.

IDI guides facilitated discussions with program beneficiaries on perceptions and social norms related to similar topics explored in the FGDs. The guide was also used to map the influence on child nutrition, child illnesses, ANC and immunization, and FP by asking about influential groups. The responses were used to create **an influence chart.** For ACG members, IDI guides explored their perspective on the ACG model and its operation. It gathered information about ACG activities, including health message dissemination and community dialogues on MNCH+N, FP, and malaria. The guide also helped in eliciting information on challenges encountered in addressing social norms.

### Data collection

Data collection activities were conducted in 3 LGAs per study state after obtaining written informed consent from each participant. The selection of these LGAs was determined by the implementation level of the ACG intervention. Fieldwork was implemented simultaneously in study states over two weeks in May 2021. Participants were recruited based on the study’s inclusion criteria with the assistance of local government health educators and community mobilizers who were familiar with the terrain and understood the fundamentals of the Breakthrough ACTION/Nigeria project. Furthermore, 12 trained field assistants worked closely with mobilizers to ensure that the appropriate participants were recruited. In all, 75 data-gathering activities were conducted across the study states, including 24 FGDs comprising of an average of 7 persons per group and 51 IDIs as shown in Table 1 below. Each activity lasted for 45–60 minutes on average.

**Table 1:** Breakdown of data gathering activities per state and gender.

Data Gathering Activities	Bauchi	Sokoto	Total
FGDs Male	6	7	13
FGDs Female	6	5	11
IDIs Male	13	15	28
IDIs Female	11	12	23
Total	36	17	75

### Data management and analysis

Data were transcribed, reviewed, properly labelled, and stored in a password-protected computer. NVivo (released in March 2022) [40] software was used to manage data throughout the analysis process. The data were analyzed using the reflexive thematic analysis steps as spelt out by Braun and Clarke [41]. Six researchers conducted an initial review to ensure initial familiarization with the data and develop a coding framework. Subsequently, transcripts were coded by four researchers with the aid of the coding framework to ensure that codes were consistently applied. The resulting discrepancies were resolved through a consensus-building approach guided by the research objectives. Codes were then organized into themes. These initial themes were reviewed and well-defined and used to generate the final themes for analysis. An influence chart per health area was developed in the study states where responses from beneficiaries were aggregated and converted into percentages, which were then presented in charts in the results section.

### Ethical considerations

The research was granted ethical clearance by the Institutional Review Board of Tulane University (Approval number: 2019–1646), the National Health Research Ethics Committee (Approval number: NHREC/01/01/2007-21/06/2020), as well as the Sokoto State Health Research Ethics Committee (Approval number: SKHREC/039/2021) and the Bauchi State Health Research Ethics Committee (Approval number: NREC/03/11/19B/2021/16). Prior to data collection, all participants in the study provided written informed consent. The study strictly followed principles of research ethics, ensuring participant autonomy and privacy, justice, beneficence, and non-maleficence.

## Results

### Participants’ demographics

A total of 51 IDIs were conducted including 26 in Bauchi State and 25 in Sokoto State. We also conducted FGDs that included 162 people in total, or 86 in Bauchi State and 76 in Sokoto State respectively. Across these interviews and discussions, most participants were married (48/51 IDIs and 135/162 FGD participants), practiced Islam (48/51 IDIs and 161/162 FGD participants) and were Hausa (21/51 IDIs and 101/162 FGD participants). More than half of the participants were male (28/51 IDIs and 97/162 FGD participants) and were 35 years old or younger (30/51 IDIs and 86/162 FGD participants). Nearly all participants had attended some formal education level with many attending at least tertiary education (25/51 IDIs and 84/162 FGD participants).

### The advocacy core group model

#### Activities of ACG

The duties of ACG members were investigated and contrasted with the descriptions outlined by the Breakthrough ACTION project as part of the ACG model design. As part of the ACG approach, ACG members were expected to facilitate SBC messaging on MNCH+N-related behaviors and create demand related services. We found evidence that aligns with these expected responsibilities. Participants also mentioned referring program beneficiaries to health facilities to access care. Community platforms routinely used in advocacy and sharing health promotion messages include town hall meetings, sermons, naming, and wedding ceremonies, and in some cases media platforms.

*In summary, we help people change harmful norms and take positive attitudes and lifestyles. For example, I have people in this area that believe since their parents grow up, they do not go to the hospital because they use native herbs for illness and childbirth and contraception. But now we have taught them to leave these norms and accept modern health care services.*

**–Women Leader, 35 yrs. old, Sokoto**

When asked about their perceived roles and responsibilities, ACG members corroborated that their primary roles and responsibilities include attempting to positively influence program beneficiaries on practices related to the priority health behaviors and supporting demand creation for MNCH+N and family planning services.

*I counsel newly married couples on the need to give space in-between birth. Also, during naming ceremonies, I try to use that avenue to educate couples that whoever does not provide for his family by taking good care of their needs is even worse than an unbeliever therefore they should give birth in a manner that they will be able to meet the demands that comes with the family.*

***–*Religious Leader, 60yrs old, Bauchi.**

#### Reach of ACG members

**Fig 1:**
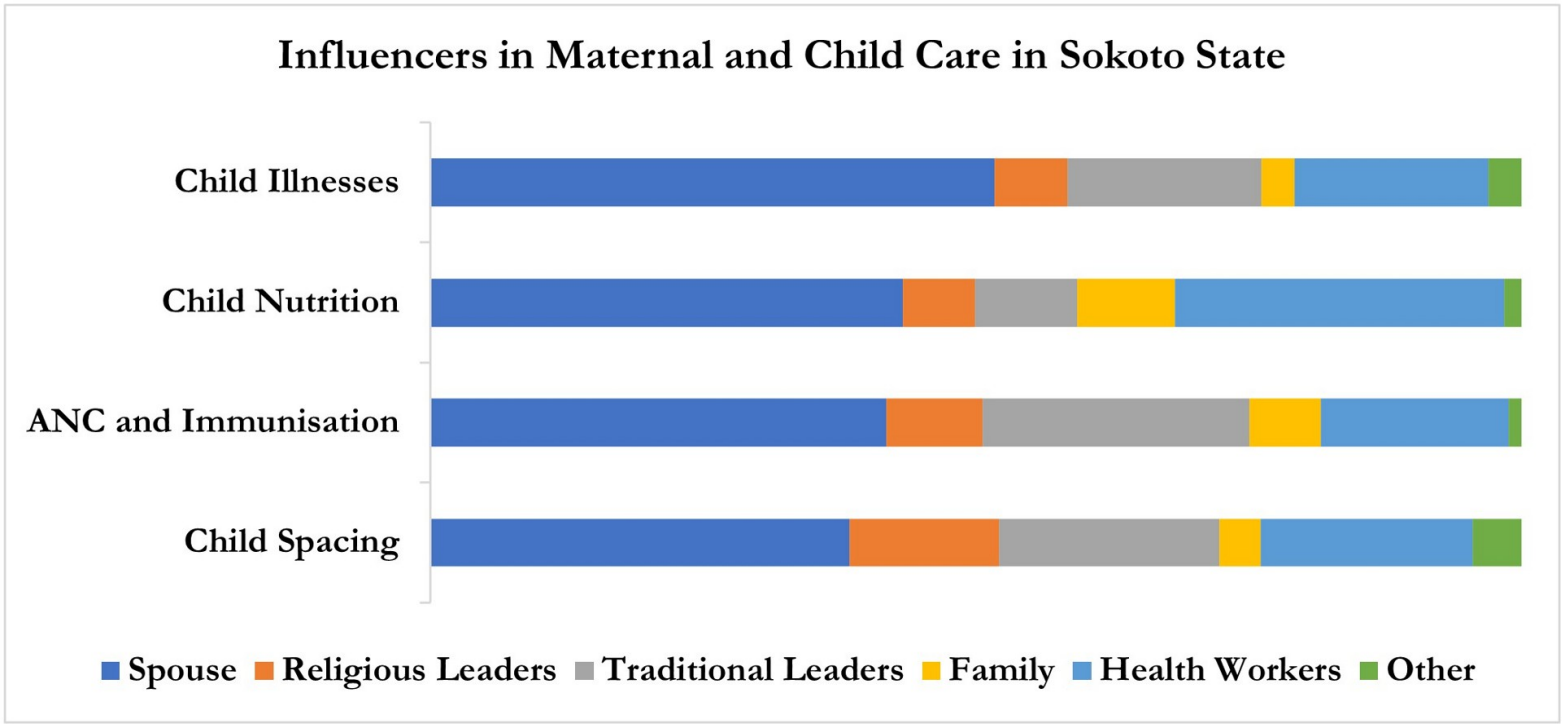
Influencers in maternal and child care in Sokoto State.

**Fig 2:**
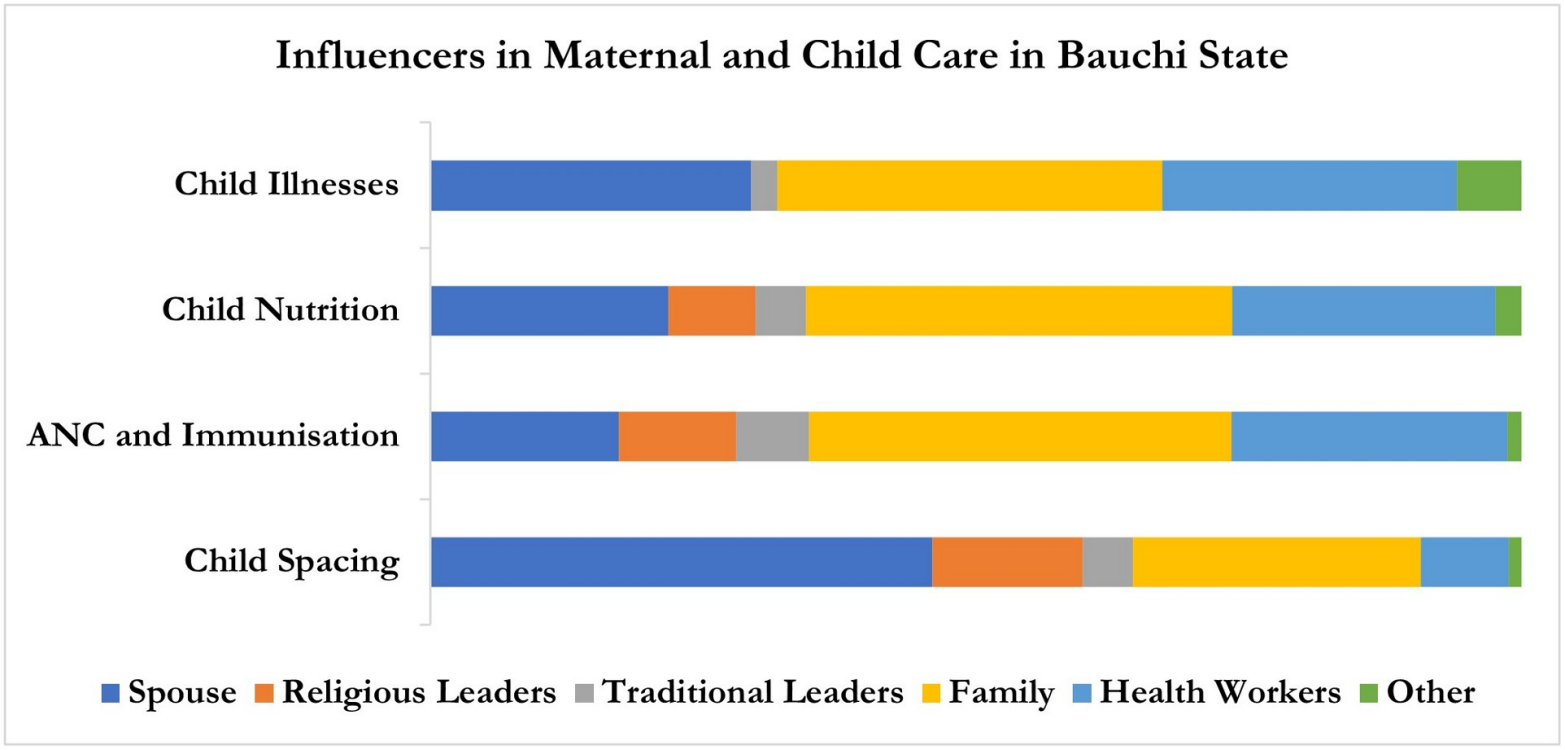
Influencers in maternal and child care in Bauchi State.

In examining the influence on beneficiaries’ health, the potential of ACGs to influence health appears strong yet can vary by geography and health topic as shown in Figs 1 and 2. Although spouses had the most influence in Sokoto state across health areas, ACGs also had a major influence. For FP, ANC and immunization, and child illnesses, the primary influencers, in order, were spouses, traditional leaders, health workers, and religious leaders. However, in the case of child nutrition, the majority of beneficiaries reported spouses and health workers as influencers, followed by a smaller number who mentioned traditional leaders, family members, and religious leaders as influencers, in that order.

In Bauchi state, family members, health workers, and spouses had the most influence compared to ACGs. The primary influencers for ANC and immunization, as well as child nutrition, were identified as family members, followed by health workers and spouses. Religious and traditional leaders were reported as influencers by only a small number of beneficiaries across these health areas. For child illnesses, a majority of beneficiaries identified family members as the main influencers, followed by spouses and health workers. In the case of FP, nearly half of the beneficiaries in Bauchi State reported spouses as the major influencers, followed by family members and religious leaders.

The comparison of influencers of maternal and childcare across the two study states shows that spouses play a pivotal role in decision-making. In addition to this, the data showed that the influence of family members is more dominant in Bauchi state. While religious leaders were moderately influential in both states, their impact was slightly more pronounced in Sokoto state. In contrast traditional leaders had minimal influence in both states.

*Potential for shifting norms*. This section presents the findings of the social norms’ exploration with program beneficiaries and ACG members, who were both asked to discuss a vignette depicting a young couple experiencing various situations related to the priority health behaviors and social norms identified by Breakthrough ACTION/Nigeria as the focal points of the ACG programming. All participants in the study expressed their opinions in reaction to the vignettes presented on health areas, including factors that influence health behavior. The findings are provided below per priority health area.

*Family Planning*. ACG members and beneficiaries responded to the vignette that FP is advantageous for both the mother’s and child’s health. Participants noted that they had been exposed to information on the benefits of FP through religious and traditional leaders, and indicated the increased knowledge has generally resulted in a positive shift in perception and behavior.

*Right now, I think, in a group of 100 [women], you can have up to 80 [women] that subscribe to the idea of family planning. Sincerely.*

–**Female Beneficiary, 25yrs old, Bauchi**

The findings also indicated that religious beliefs influence contraceptive use. An example of this was the belief expressed by a few participants that family planning is an attempt to avert birth and is contrary to religious principles. Participants claimed that some religious leaders preach against it. Nonetheless, both ACGs and program beneficiaries endorsed a positive perception and attitude towards family planning.

*You see, the issue is there are some [community leaders] that would say things like you should not use child-spacing [family planning] even before giving birth—that it is not good. They are taking it negatively, thinking it is not religiously right.*

**–Male Beneficiary, 23yrs old, Sokoto**

Overall, participants linked the ACG activities to improvements in their communities’ health behavior. According to participants, family planning has been reported to be very well accepted in areas where ACG members are operating, even among remote populations and they felt more women now utilize family planning services.

*In the past, you would find 2 to 3 wives in a household and all of them give birth in a year but with the sensitization on child spacing [FP] there are a lot of changes, they now practice child spacing in the community.*

**–Female Community Leader, 62yrs old, Bauchi**

*Antenatal Care*. When asked if the community norms influenced women’s desire and ability to attend ANC, participants responded that some women preferred traditional medicine to visiting health facilities. However, this norm was perceived to be prevalent in remote communities.

*Our people in the interior villages, you have to always include them in the sensitization, the challenge [however] is they don’t always do as they are told. They still do not agree on the importance of allowing pregnant women to go for ANC despite the sensitization. They do not prioritize visiting a health facility, they give their pregnant women herbs, they only go to a health facility when there is a complication like excessive bleeding that cannot be managed at home.*

***–*Female Beneficiary, 32yrs old, Bauchi.**

Further conversations during the vignette about the benefits of attending ANC revealed that program beneficiaries were aware of the potential risks associated with non-attendance. Also, while pregnant women in their communities understood the benefits of ANC, they were reluctant to access care without permission from husbands or influential family members.

There is the possibility of improved behavior following increased knowledge and favorable attitude towards ANC and nutritional behaviors, as favorable changes were reported by both categories of participants.

*People keep coming to the hospital, this has even increased the number of people, so far even our data increased of those women coming for antenatal routine checks.*

**–Male Religious Leader, 62yrs old, Sokoto.**

*Immunization*. When discussing issues related to immunization, program beneficiaries mentioned that it was typical to have some hesitation towards immunization, specifically because of the associated side effects. This resulted in partial and non-immunization of many children within the communities. Other normative reasons for non-immunization were beliefs that immunization led to future infertility of the child and the religious notion that ultimate protection from diseases could only be provided by God.

*People are sometimes educated but reluctant to take up immunization because of religion, they will say things like ‘God will protect’.*

**–Female Beneficiary, 24yrs old, Bauchi.**

*Exclusive Breastfeeding and Child Nutrition*. Participants concurred that children needed a well-balanced diet to stay healthy. Yet, different individuals had varying ideas about what constituted quality nutrition for children. While many people recognized EBF as an important practice for raising healthy children, it was unclear whether the exact meaning of EBF was understood. The findings show that some prevailing breastfeeding norms still exist within communities. For instance:

In Sokoto, infants are given holy water from pilgrimage (*Ruwan Zamzam*) after delivery.

*Here in our community when a person is delivered of a child, the father comes with a special kind of drink for the mother and child. It is like pure [holy] water from Saudi Arabia blessed by the Prophet Mohammed.*

**–Male Beneficiary, 25yrs old, Sokoto**

EBF is not accepted in some communities because they believe it is insufficient for the child’s growth. Also, Infants are denied the first breastmilk with the assumption that the colostrum is contaminated and has no benefit.

*There is tradition. Some say when a woman gives birth, the breast needs to be washed before she breastfeeds. Then to some, until they bring the milk of sheep, camel, or goat to the baby, before giving the mother’s breast.*

**–Male Beneficiary, 24yrs old, Bauchi.**

#### Gender norms: Unequal decision making

ACG members and program beneficiaries’ reactions to the vignettes regarding decision-making revealed that women lack the autonomy to make health decisions and independently seek care which reflected uneven power dynamics. According to some younger female beneficiaries, even if a woman has her own money to pay for health care, she must still seek her husband’s approval for treatment. The need to obtain approval to seek healthcare likely results in negative impacts on healthcare access, as described by participants relating stories of people they recalled.

*If she goes to the facility, her marriage is at stake, she cannot decide whether the child is healthy or sick, the marriage will collapse.*

**–Traditional Leader, 35yrs old, Sokoto**

When husbands were unavailable, mothers-in-law were also significant determinants of health-seeking behavior for women on their health and that of their children.

*If a man is travelling; he leaves the responsibility of health decision making for his mother.*

**–Women Leader, 35yrs old, Sokoto.**

ACG members and beneficiaries perceived that husbands/male guardians are increasingly likely to grant “advanced permission” for women to seek necessary healthcare. Furthermore, it was considered acceptable for women to seek care in case of extremely urgent conditions or during a life-or-death emergency.

*If a serious situation that require swift response comes up, and the husband is absent a woman can go ahead to save the situation without necessarily waiting to consult.*

**–Male Beneficiary, 30yrs old, Bauchi**

#### Consequences for women making health decisions

The expectation to seek approval from spouses or mothers-in-laws (when husbands were not present) was described as an injunctive norm, and failure to act within this norm resulted in sanctions which includes community reprimands and the possibility of divorce.

*She might actually pass through a lot of beatings from her husband and even lose her marriage.*

**–Female Beneficiary, 27yrs old, Bauchi**

Participants were further asked to comment on violence as a consequence of ‘disrespect’ for the authority of the man on health decisions. Many considered violence as a correctional measure. This authority is grounded in the perception that men are the providers for the household.

*The man is the burden bearer as everything is on his shoulders, and all the needs of the house, so his wife is under him, and so I have the right to keep on insisting over an idea I do not like… I do not see slapping my wife as an issue rather, it will serve as deterrent to other women who want to behave like her.*

**–Male Beneficiary, 31yrs old, Bauchi**

Lack of exposure and education contributes to the violence towards women. In addition, violence was perceived to be more widespread among young males and those in the early stages of marriage than among the elderly.

*It is few men that will agree that beating their wives is good or justify hitting a woman. I think it is most common with young boys that just got married. If there are elders or religious leaders there, they will condemn such an attitude and caution him.*

**–Female Beneficiary 24yrs old, Sokoto**

Contrarily, participants mentioned that some people in the community have become more progressive in their beliefs and were likely to condemn violent behaviors.

*Plenty people in this community will think that he does not know what he is doing [if he beats his wife] because people are educated now and if the lady leaves his house, no woman will want to marry him again because they conclude that he is a wife beater.*

***–*Female Beneficiary, 21yrs old, Sokoto**

*Challenges to the ACG efforts to address norms*. Our findings show that several challenges have contributed to the efforts of ACGs to influence norms. Many ACG members complained that despite several sensitization and awareness efforts, broader health systems challenges such as the poor service received at facilities, negative attitude of health workers, and insufficient health workers discourage women from accessing care.

*Due to awareness on a regular basis, there has been progress sincerely. The problems we have now is between the people and the hospitals, issues like limited health workers, when a woman is in labor, and she goes to the hospital she does not get to see the workers.*

**–Women Leader, 48yrs old, Sokoto**

Another challenge was the socioeconomic status of program beneficiaries. In both states, community leaders noted that changing the behavior towards MNCH was easier than convincing a woman who can barely afford her basic needs to visit a health center when services were not free-of-charge.

*For example, if a woman in the village gives birth and immunization is to commence, you see she cannot come on foot. That was why I showed you that there is poverty. This common 50 naira for transport fare, will rather be used for Maggi [connotation for food], isn’t it?*

**–Female Beneficiary, 25yrs old, Bauchi**

Some male ACGs have expressed hesitancy in engaging communities in discussions about certain health issues, such as family planning. This reluctance stems from their perception of potential discomfort, particularly when addressing these matters with female program beneficiaries.

*It is difficult to gather married women and begin to mention sex and blood that comes out of them etc. Imagine mentioning condoms. But talking about the easy ones… of course, it does not take a lot to talk about vaccination and going for antenatal*

**–Male Community Leader, 60yrs old, Bauchi.**

When asked what priority health behaviors were difficult to discuss, family planning and breastfeeding norms also appeared to be the most challenging topics across all categories of ACG members. Further conversation disclosed that for child-spacing the reasons were rooted in religion.

*Family planning is sometimes difficult because there are religious and traditional aspects. A leader cannot come to you directly and tell you not to give birth, or you should stop her from giving birth. It is difficult for them because it is supposed to be personal.*

**–Male Youth Leader, 23yrs old, Bauchi**

There may also be a perception by ACG members that they do not have tangible benefits to offer communities who they are reaching on health topics. The ability to offer incentives appears to be something the members feel could strengthen their advocacy efforts.

*Sometimes we tell people to meet us, but we have nothing to give the people in the meeting, not even us the leaders in ACG, but those traditional leaders, and the other leaders we invited.*

**–Women’s Leader, 48yrs old, Sokoto**

## Discussion

This study qualitatively explored the operation of and potential for effectiveness of the ACG model in influencing community-level norms and individual MNCH+N, FP, and malaria prevention behaviors. Overall findings from this study indicate that as a result of the ACG activities, participants reported increased awareness of key health behaviors. Furthermore, data revealed the potential to influence and modify existing norms.

ACG members described achievements in community engagement, and linkages with healthcare facilities through direct engagement with program beneficiaries across a range of activities, such as community religious events and ceremonies, household visits, and community dialogues. This finding is consistent with what has been documented in other studies. For example, a study on complementary feeding in Kaduna state, Nigeria showed that parents were reached with messages on complementary feeding through trained religious leaders and community-based organizations using religious gatherings which in turn led to improved complementary feeding practices [42]. ACG members reported perceptions of positively influencing program beneficiaries on all practices related to priority health behaviors, as well as on demand creation for MNCH+N-related services. Conversations with program beneficiaries corroborated this perception with phrases such as “we have been enlightened,” and “this no longer occurs” when discussing vignettes in the key MNCH+N areas.

We found that some norms appear to be shifting to facilitate MNCH+N-related behaviors. Although some norms, particularly those associated with EBF, may be slower to change due to their deeply ingrained nature. For example, infants are often given holy water, denied colostrum, and many individuals believe that breast milk is insufficient. This finding is consistent with several studies conducted in Nigeria, India and Nepal, where it was reported that colostrum was perceived to be impure, and could harm the infant [43–46] This is in contrast to well-known and accepted recommendations that is nutritionally adequate except when the mother is deficient [47]. While ACGs revealed that they encourage EBF for the first six months of life, the precise definition of “exclusive” used was sometimes unclear. This is a gap that could affect the effectiveness of health messaging and could relate to why breastfeeding norms remain pervasive.

Slow progress in child-bearing norms was also tied to health decision making practices. Customarily, husbands are the final decision makers for many health behaviors, including in the aspect of family planning. There was a wide-spread belief among study participants that it would be a wife’s responsibility to persuade her husband of the need for MNCH+N health services, possibly by enlisting the support of mothers-in-law, and community and religious structures. This is in line with the findings of Ahuru (2021) who shared that the contributions of women to household decisions are minimal because men are predominantly in charge of economic resources and as a result, they dictate where, when, and how their wives use health services [48]. The perceived obligation of women to persuade their male partners of the need for health care may be linked to the belief that women are subject to uneven power dynamics in households. This could yet provide a vital opportunity for improving participation in decision making through social and behavioral change programs and eventually increased autonomy for women.

ACG members were reportedly fulfilling their responsibilities by fostering discussion on collaborative decision making between spouses on health matters. This was linked to perceptions of shifts towards more shared decision making, notably in ANC and facility-based deliveries. Joint decision making has been shown to improve health outcomes, for example where women who participated in their own healthcare decisions were 33% and 20% more likely to have a minimum of four ANC visits and deliver their babies in health facilities, respectively [48]. Another dimension of ‘improved’ decision making seen in this study was the fact that husbands were reported to be increasingly likely to grant “prior consent" for their wives to seek necessary healthcare. While this could be viewed as a change in the timing of permission, this might potentially be the beginning of a more significant shift in the decision-making norm.

In this study, some male ACG members described greater comfort in discussing immunization and shied away from topics such as FP, particularly with married women. This gendered pattern could be as a result of the entrenched gender norms in northern Nigeria, particularly as there is already a reluctance to discuss related sexuality issues [49]. The reluctance of men to have these conversations could be attributed to cultural sensitivities, in which some people consider discussions about FP as inappropriate or contradictory to religious teachings, making it uncomfortable to broach the subject. Also, our finding could be because ACG members themselves share in these norms as they are members of these communities. Encouraging male involvement in family planning is crucial to address the issue. Numerous studies have demonstrated the positive impact of male engagement, particularly in addressing women’s reproductive needs, such as family planning. This involvement has been shown to positively influence the uptake of family planning methods. [50, 51].

Although the ACG program was deliberate to include women religious and community leaders to disseminate information within the community, it is important to recognize that, due to entrenched social norms, only a small number of women hold positions of authority within these communities.

Hence, until there is increased participation of women in leadership positions, male community and religious leaders will continue to be responsible for sensitization and awareness efforts. This suggests the need for more capacity building efforts targeted at male leaders to improve MNCH behavior.

Another key norm identified in this study was tolerance for GBV. GBV towards women has long been accepted as a correctional measure for ‘disrespect’ for male authority. Disrespect was perceived as a wife’s refusal to accept a husband’s perspectives, including those on health. Several studies have noted that participants described gender-based violence as an acceptable consequence towards perceived disobedience, particularly regarding decisions related to family planning. For example, the authors of a research on GBV coverage in Uyo, Nigeria, stated that there is a profound cultural notion in Nigeria that hitting a woman and disciplining a spouse is socially acceptable [52]. Community norms related to GBV appear to be shifting due to modernization and progressive beliefs. One example is the observation of ACG beneficiaries that incidents of GBV have reduced owing to increased education among program beneficiaries.

Non-normative threats to the ACG model identified in this study were associated with broader health systems issues, such as the poor health services, service providers’ attitudes, and insufficient health workforce. ACG members argued that it was already challenging to encourage women to change their health behaviors, and so women being faced with issues like poor services when they eventually visit facilities added difficulty to their work. This is consistent with the findings of a systematic review of attitudes and behaviors of maternal health care providers in interactions with clients that revealed a broad range of negative attitudes and behaviors affecting patient satisfaction with care and care-seeking such as unavailability of providers, poor communication, and authoritarian or frightening attitudes [53]. Furthermore, recent studies have also identified insufficient workforce as a challenge in selected MNCH+N services in sub-Saharan Africa [54]. These findings suggest that behavior change interventions cannot be implemented in isolation and may need to be accompanied by interventions aimed at addressing supply-side health system issues.

### Limitations

Given the qualitative nature of this study, it was not possible to objectively measure changes in social norms. Understanding social norms such as those identified in this report is key for social behavior change research and its potential to inform programming. Wallen and Romulo describe a social norm as: “a normative social belief, which is an individual’s beliefs about the behaviors and evaluations of others in a social setting: that is, a cognitive construct and mental representation of the actual social norm” [55]. Caution is required in presuming that changes in norms automatically result in changes in behavior [56].

## Conclusion

Our findings indicated that the ACG model was linked to increased awareness of health issues across key priority health areas and improvements in the uptake of recommended MNCH+N behaviors. There is a need for the model to continue targeting norms that are slow to change such as those related to EBF and GBV. This could mean more capacity building for ACGs to further help drive social and behavior change. Furthermore, the prospect of ACG members holding identical normative ideas that limit MNCH+N behavior uptake necessitates interventions in value clarification and attitudinal adjustment. Increased and concerted interventions to address health system issues will further complement the efforts to drive behavior change.
